# Scoping review of e-cigarette use in the perioperative setting: a protocol

**DOI:** 10.1136/bmjopen-2026-118679

**Published:** 2026-05-24

**Authors:** Suzanne R Harrogate, Jonathan Livingstone-Banks, Robert Hinchliffe, Ian Pope, Ronelle Mouton

**Affiliations:** 1 Department of Anaesthesia, NHS North Bristol NHS Trust, Bristol, UK; 2 Centre for Evidence-Based Medicine, University of Oxford, Oxford, UK; 3 Health Sciences, University of Bristol, Bristol, UK; 4 Department of Vascular Surgery, NHS North Bristol NHS Trust, Bristol, UK; 5 Norwich Medical School, University of East Anglia, Norwich, UK

**Keywords:** Vaping, electronic nicotine delivery systems, perioperative care, anesthesiology, surgery

## Abstract

**Abstract:**

**Introduction:**

E-cigarette use (often referred to as ‘vaping’) has increased rapidly over the past decade. Tobacco smoking is a well-established risk factor for adverse perioperative outcomes. While UK guidance supports e-cigarette use as a harm reduction and smoking cessation strategy, the perioperative implications of e-cigarette use are unclear. This scoping review aims to map the breadth and nature of the available evidence on e-cigarette use in the perioperative setting. It will describe how perioperative e-cigarette use is defined and measured, identify the perioperative populations and settings which have been studied, summarise reported perioperative outcomes and identify key knowledge gaps that should be addressed in future research.

**Methods and analysis:**

This review will be conducted in accordance with Joanna Briggs Institute methodology and reported according to Preferred Reporting Items for Systematic Reviews and Meta-Analyses Extension for Scoping Reviews (PRISMA-ScR) guidelines. MEDLINE, EMBASE, CINAHL, PsycINFO, Cochrane Central, Web of Science and grey literature sources will be systematically searched from 2003, when the first commercially available e-cigarette was introduced, to February 2026. Studies will be screened, and data extracted by two independent reviewers. Studies of any design examining perioperative e-cigarette use in the perioperative period, across all surgical specialities, will be included. Data will be synthesised narratively and presented using tabular and visual summaries. The study will be undertaken between 9 February and 1 August 2026.

**Ethics and dissemination:**

Ethical approval is not required for scoping reviews. Findings will be disseminated by conference presentation and publication in a peer-reviewed open-access journal and communication with stakeholders.

**STRENGTHS AND LIMITATIONS OF THIS STUDY**

- A scoping review is the most appropriate methodology to systematically map the breadth and nature of available evidence on vaping in surgical populations.
- This protocol adheres to best practice scoping review methodology, including a comprehensive, librarian-assisted search strategy, to ensure methodological rigour.
- Terminology for e-cigarettes is variable and has evolved rapidly alongside the technology, which may limit search completeness despite use of a published ontology and full synonym list.

## Introduction: rationale

 An e-cigarette is an “electronic vaping device that is hand held and produces for inhalation an aerosol formed by heating an e-liquid using a battery-powered heating coil.”^1^ E-cigarettes are often colloquially referred to as ‘vapes’ but, as this term is technically less precise, ‘e-cigarette’ is the recommended term in the addiction literature.^1^ The prevalence of e-cigarette use among English adults has risen rapidly over the past decade: from 1.3% in 2013, to 10% in 2023.^2^ Although it remains most common in people who currently or formerly smoke, there is a large and growing cohort who use e-cigarettes despite never having regularly smoked combustible tobacco. Growth has been especially marked in young adults, with a sharp rise since 2021 coinciding with the widespread availability of disposable devices. In response to concerns about youth uptake and environmental impact, the UK government implemented a nationwide ban on the sale of disposable e-cigarettes in 2025.^3^ Currently, 22.7% of 18-year-olds report long term e-cigarette use, defined as current use for a period of more than 6 months.^2^ With increasing overall prevalence, it is likely that a growing proportion of surgical patients use e-cigarettes, making it important to understand the perioperative implications.

Tobacco smoking is a well-established risk factor for adverse perioperative outcomes,^4 5^ and the benefits of preoperative smoking cessation are supported by a substantial evidence base,^6^ with each week of preoperative abstinence decreasing the risk of complications by 19%.^7^ UK guidance from the National Institute for Health and Care Excellence and the Royal College of Physicians supports e-cigarette use as a harm reduction strategy^8^ and as an aid towards smoking cessation.^9^ Evidence, including a pilot trial in the perioperative setting, suggests e-cigarettes are a popular and effective aid for smoking cessation.^10 11^ Nevertheless, there are plausible mechanisms by which inhaled nicotine and e-cigarette vapour could lead to perioperative complications^12^,^14^ and literature reviews in specific surgical specialities suggest a possible association with complications.^15 16^

The extent, nature and characteristics of the evidence relating to e-cigarette use in the perioperative period are unclear. This scoping review will systematically map the available literature, identify key concepts and knowledge gaps, and clarify the range of outcomes and study designs that have been investigated. The findings will inform the feasibility and design of future research and support the development of evidence-based perioperative practice and policy.

### Objectives

1. To identify, describe and map the available evidence from studies that explore perioperative e-cigarette use and its consequences.
2. To identify potential gaps in knowledge around perioperative e-cigarette use.
3. To identify whether a systematic review would be possible based on the characteristics of the available evidence.

## Methods

The scoping review will be undertaken using the methodological framework of the Joanna Briggs Institute (JBI)^17^ and reported according to the Preferred Reporting Items for Systematic Reviews and Meta-Analyses Extension for Scoping Reviews (PRISMA-ScR).^18^ This scoping review is registered with Open Science Framework (study identifier: 5fqc3).

### Information sources and search strategy

The search strategy will be designed in collaboration with an experienced academic librarian. A draft search strategy is available as online supplemental material. The design will consider the accepted ontology for tobacco, nicotine and vaping products, and include all listed synonyms for e-cigarette produced in this team’s previous scoping exercise.^1^ Less commonly used synonyms (such as ‘disposables’, ‘puff bars’, ‘Elf bars’ and ‘pod mod’) will not feature in the search strategy. Relevant studies using these terms are likely to include clarifying terminology using more conventional terms such as ‘e-cigarette’ or ‘vape’ and will therefore be captured by our search.

The search will be undertaken from 2003, when the first commercially available e-cigarette was introduced,^19^ to February 2026. The search will use the following information sources: CINAHL (Ebsco), EMBASE (Ovid), MEDLINE (Ovid), PsycINFO (Proquest), Cochrane CENTRAL (Cochrane) and Web of Science (Clarivate). A grey literature search will also be undertaken, including clinical trial registries (clinicaltrials.gov, WHO international clinical trials registry platform and the EU clinical trials register) and repositories of dissertations and theses (proquest dissertations and theses global). This manual search will be supported by the R package greylitsearcher (Zenodo 2022).^20^ Forward and backward citation chasing of included full text papers will be undertaken manually, supported by the R package citationchaser (Zenodo 2021).^21^

### Study selection and eligibility criteria

All study types in humans will be included, including qualitative studies, non-randomised and retrospective reports, case reports and other non-comparative studies. There will be no restriction on the country of study or publication language.

Studies will be included if they meet the following criteria.

#### PCC framework

##### Population

Humans of any age who use e-cigarettes and are undergoing a surgical procedure or a procedure necessitating anaesthesia of any form (general, regional and local). This includes obstetric patients who have undergone a surgical intervention (including repair of any grade of perineal tear using sutures, whether performed in the operative theatre or elsewhere), but excluding analgesic only interventions (such as patient controlled analgesia and epidural analgesia for labour). Studies that include a planned subgroup of patients who use e-cigarettes will be included.

##### Concept

Use of e-cigarettes in the perioperative period. This includes exclusive e-cigarette use, dual use alongside cigarettes and both nicotine-containing and nicotine-free e-cigarettes.

##### Context

The perioperative period, in both inpatient and outpatient settings. Any study type, published worldwide, in any language.

### Definitions

We will use the following definitions in our writing (table 1), but for the purpose of inclusion in the scoping review we will accept the definitions and terms used by the retrieved studies. This will be compared with the published ontology and commented on as an outcome of the scoping review.

**Table 1:** Definitions of terms relating to e-cigarette use and the perioperative period

Term	Definition
Perioperative	Defined according to the Centre for Perioperative Care: “from the moment surgery is contemplated through to full recovery”.^24^
Smoking Synonyms: tobacco smoking	Defined according to the WHO: “Inhalational tobacco use (including smoking cigarettes, cigars, cigarillos, pipe tobacco and other forms of inhalational tobacco”.^25^
Nicotine replacement therapy	Nicotine product that aims to reduce motivation to smoke and the withdrawal symptoms often experienced during an attempt to stop smoking, and therefore increase the likelihood of remaining abstinent.^26^
Abstinence	A period in which there is no use of combustible cigarettes.^27^ We included abstinence assessed by any method, including self-reporting and biochemical verification.
Definitions for terms related to e-cigarettes, products and devices have been drawn from the ontology published by Cox and colleagues in 2023.^1^ The most relevant of these are listed below.	
E-cigarette Synonyms: (terms that are often used instead of the formal label) Electronic cigarette Vape Vaping device Nicotine vaping device Alternative nicotine vaping device Electronic nicotine vaping device Electronic vaping device Alternative nicotine delivery system ANDS Electronic nicotine delivery system ENDS Vaping product Nicotine vaping product NVP	An electronic vaping device that is hand held and produces for inhalation an aerosol formed by heating an e-liquid using a battery-powered heating coil.^1^
Vaping device	A product that produces a vapour for inhalation by a person.^1^
Electronic vaping device	A vaping device that produces vapour by electrically heating a substance that is an e-liquid or a solid.^1^
Nicotine-containing e-cigarette	An e-cigarette whose e-liquid contains nicotine.^1^
Combustible tobacco-containing product	A tobacco-containing product in which the tobacco is processed to make it suitable for burning for the user to ingest the smoke.^1^
Cigarette	A combustible tobacco-containing product that is a thin cylinder of finely cut tobacco encased in a thin paper.^1^
Inhaled nicotine-containing product	A nicotine-containing product that is manufactured to enable users to draw a nicotine-containing vapour into the respiratory tract so that nicotine can be absorbed through the lining of this tract.^1^

ANDS, alternative nicotine delivery system; ENDS, electronic nicotine delivery system; NVP, nicotine vaping product.

### Study records

#### Data management

EndNote (Clarivate, 2024) will be used for reference management and deduplication prior to screening. The software Rayyan (Rayann, 2016)^22^ will be used for subsequent citation management and screening. Data will be managed using Microsoft Excel (Microsoft Corporation, 2024).

#### Screening and selection process

Two blinded reviewers will independently review the title and abstracts of all citations identified by the search strategy against the above inclusion criteria. After removing blinding, disagreements will be discussed with input from senior authors until a consensus is reached. Two blinded reviewers will then undertake independent full text screening, with disagreement resolved in the same way. Screening of citations and full texts published in any non-English language will be undertaken with the aid of the translation program Google Translate (Alphabet Inc.), with assistance sought from professional translation services if required.

#### Data extraction

Data will be extracted by one reviewer and verified by a second. Data will be collected using a standardised form (online supplemental material) and entered into Microsoft Excel (Microsoft Corporation, 2024). It is recognised that in scoping reviews, data extraction is an iterative process.^23^ The review team will meet at regular intervals to discuss the data extracted and the narrative synthesis as it progresses. During this process, it may become apparent the authors that additional data items are of interest. Extraction of data items that were not prespecified will be described and rationalised in the final report.^23^ Data extraction from texts published in any non-English language will be undertaken with the aid of the translation program Google Translate (Alphabet Inc.), with assistance sought from professional translation services if required.

Where appropriate, the corresponding author of included studies will be contacted to discuss and request any missing or unclear data items.

#### Data items

Data to be extracted will include

- Study design.
- Participant demographics.
- Characteristics of e-cigarette and tobacco use reported.
- Study outcomes.
- Study results.
- Conclusions.
- Stated limitations.

### Data synthesis and analysis

The search results and the process of study selection will be reported in a PRISMA flow chart.^18^ Studies will be analysed inductively, including a basic qualitative content analysis. Where appropriate, results will be presented as a narrative synthesis, supported by tabular and visual summaries as appropriate.

We will record and report on all case reports that meet our inclusion criteria and provide a narrative overview of the case reports in our results. We will, however, only report in detail on the specifics of case reports which we feel bring useful information to the discussion (eg, those which report respiratory complications).

An integrated consultation phase will be adopted where the initial results and data extracted are reviewed and discussed with senior members of the research team, including experts in smoking cessation intervention (IP, JL-B), anaesthesia (RM) and surgery (RH). In keeping with established scoping review methodology, this iterative process will guide additional data extraction from papers as deemed necessary.^17^

### Patient and public involvement

The research question was developed using feedback from people with lived experience of smoking tobacco and undergoing surgery, who were consulted to inform a proposed trial of a smoking cessation intervention in people undergoing vascular surgery.

## Ethics and dissemination

Ethical approval is not required for scoping reviews.

The study will be disseminated by conference presentation, and publication in a peer-reviewed open access journal. We will share the results with patient partners and members of the public and the charitable stakeholder, Action on Smoking and Health.

## Supplementary material

10.1136/bmjopen-2026-118679 online supplemental file 1

10.1136/bmjopen-2026-118679 online supplemental file 2
