# Supplementary material for: Scoping review of e-cigarette use in the perioperative setting: a protocol
Source: BMJ Open. 2026 May 24;16(5):e118679. doi: 10.1136/bmjopen-2026-118679 (PMC13202153; doi:10.1136/bmjopen-2026-118679)
Supplement: online supplemental file 1 [file bmjopen-16-5-s001.docx]

**MEDLINE Search Strategy**

|  | Search |
| --- | --- |
| 1 | Vaping/ |
| 2 | Electronic Nicotine Delivery Systems/ |
| 3 | E-Cigarette Vapor/ |
| 4 | vape*.ti,ab,kw,kf,mp. |
| 5 | vaping.ti,ab,kw,kf,mp. |
| 6 | electronic nicotine.ti,ab,kw,kf,mp. |
| 7 | (vapo?r* adj3 nicotine).ti,ab,kw,kf,mp. |
| 8 | ecig*.ti,ab,kw,kf,mp. |
| 9 | e-cig*.ti,ab,kw,kf,mp. |
| 10 | electronic cigarette*.ti,ab,kw,kf,mp. |
| 11 | electronic-cigarette*.ti,ab,kw,kf,mp. |
| 12 | nicotine vaping product*.ti,ab,kw,kf,mp. |
| 13 | electronic nicotine delivery system*.ti,ab,kw,kf,mp. |
| 14 | (ENDS adj3 nicotine).ti,ab,kw,kf,mp. |
| 15 | (NVP adj3 nicotine).ti,ab,kw,kf,mp. |
| 16 | (ANDS adj3 nicotine).ti,ab,kw,kf,mp. |
| 17 | alternative nicotine delivery system*.ti,ab,kw,kf,mp. |
| 18 | non-tobacco nicotine.ti,ab,kw,kf,mp. |
| 19 | non tobacco nicotine.ti,ab,kw,kf,mp. |
| 20 | nontobacco nicotine.ti,ab,kw,kf,mp. |
| 21 | non-combustible cigarette.ti,ab,kw,kf,mp. |
| 22 | exp Perioperative Care/ |
| 23 | exp Perioperative Period/ |
| 24 | exp Anesthesiology/ |
| 25 | exp Anesthesia/ |
| 26 | exp Specialties, Surgical/ |
| 27 | exp Surgical Procedures, Operative/ |
| 28 | exp Intraoperative Complications/ |
| 29 | exp Postoperative Complications/ |
| 30 | (peri-operativ* or perioperativ*).ti,ab,kw,kf,mp. |
| 31 | (pre-operativ* or preoperativ*).ti,ab,kw,kf,mp. |
| 32 | (post-operativ* or postoperativ*).ti,ab,kw,kf,mp. |
| 33 | (intra-operativ* or intraoperativ*).ti,ab,kw,kf,mp. |
| 34 | an?esthe*.ti,ab,kw,kf,mp. |
| 35 | (surger* or surgical).ti,ab,kw,kf,mp. |
| 36 | (operation or operativ*).ti,ab,kw,kf,mp. |
| 37 | 1 or 2 or 3 or 4 or 5 or 6 or 7 or 8 or 9 or 10 or 11 or 12 or 13 or 14 or 15 or 16 or 17 or 18 or 19 or 20 or 21 |
| 38 | 22 or 23 or 24 or 25 or 26 or 27 or 28 or 29 or 30 or 31 or 32 or 33 or 34 or 35 or 36 |
| 39 | 37 and 38 |
