# Supplementary material for: Scoping review of e-cigarette use in the perioperative setting: a protocol
Source: BMJ Open. 2026 May 24;16(5):e118679. doi: 10.1136/bmjopen-2026-118679 (PMC13202153; doi:10.1136/bmjopen-2026-118679)
Supplement: online supplemental file 2 [file bmjopen-16-5-s002.docx]

| **Study demographics** | |
| --- | --- |
| Study ID |  |
| Author |  |
| Year of publication |  |
| Country |  |
| Healthcare setting |  |
| Study design  *RCT, cohort, case-control, case series, narrative review*  *multiple or single site* |  |
| Study methods  *Ethics*  *Randomisation*  *Follow up* |  |
| Aim / research question |  |
| **Population Characteristics** | |
| Surgical population  *day case/not*  *surgical speciality*  *elective/emergency* |  |
| Sample size |  |
| Intervention/cohort and comparator groups  *Cessation advice or intervention given if any* |  |
| Patient age, sex, comorbidities and other reported demographics |  |
| Smoking status categories  *What they are (e.g. current, previous, never, dual)*  *How they are defined*  *Biochemically assessed or patient reported* |  |
| E-cigarette use status categories  *What they are (e.g. current, previous, never, dual)*  *How they are defined*  *Biochemically assessed or patient reported* |  |
| **Characteristics of e-cigarette use reported** | |
| Device features  *type of device (open system, pod device, closed system), resistance of the atomiser*  *e-liquid nicotine concentration, e-liquid flavouring* |  |
| E-cigarette “dose”, and how this was calculated  *Intensity of use, duration of use, chronicity*  *Biochemically assessed or patient reported* |  |
| Perioperative vaping timing  *on the day*  *cessation interval* |  |
| **Outcomes** | |
| Published outcomes  *Definition*  *Biochemical or patient reported* |  |
| **Study results** | |
| Loss to follow up |  |
| Results and direction of effect |  |
| Statistical significance |  |
| **Conclusions** | |
| Author’s interpretation/study conclusions |  |
| **Limitations** | |
| Authors’ stated limitations |  |
| Authors’ recommendations for further work |  |
| **Discussion points of interest** | |
|  | |
