# Supplementary material for: Scoping review of e-cigarette use in the perioperative setting: a protocol
Source: BMJ Open. 2026 May 24;16(5):e118679. doi: 10.1136/bmjopen-2026-118679 (PMC13202153; doi:10.1136/bmjopen-2026-118679)
Supplement: Supplementary file 3 [file bmjopen-2026-118679.reviewer_comments.pdf]

PEER REVIEW HISTORY

BMJ Open publishes all reviews undertaken for accepted manuscripts. Reviewers are asked to complete a checklist review form and are provided with free text boxes to elaborate on their assessment. These free text comments are reproduced below.

ARTICLE DETAILS

Title (Provisional)

Protocol for a scoping review of e-cigarette use in the perioperative setting

Authors

Harrogate, Suzanne R; Livingstone-Banks, Jonathan; Hinchliffe, Robert; Pope, Ian; Mouton, Ronelle

---

VERSION 1 - REVIEW

**Reviewer**	**1**
**Name**	**McKee, Martin**
**Affiliation**	**London School of Hygiene & Tropical Medicine**
**Date**	**01-Apr-2026**
**COI**	**None**

This is a timely proposal, given the growing evidence of harm associated with e-cigarettes (including a recent review of their carcinogenic effects). And, as the authors note, given the strong evidence of harm associated with pre-operative smoking, the case is well made. One problem with following the increasingly strict formulaic approach to reviews is that the questions may be answered but leave as many questions as it answers. In this case, I think it would help to extract whatever information is in the studies on biological mechanisms. This information would need to be combined with evidence from studies that would not be included in this search, but would help to explain why any effects were seen. This could then be explored in the discussion section of the final review. But maybe this goes beyond the very formulaic guidelines for reviews.

I'm sure the editors will have checked that the protocol complies with all their requirements, such as describing planned or ongoing reviews, not including data, contain a detailed rationale, and transparent methods. For the sake of completeness, I at least feel that the authors have done all this.

There are only a few things that may need to be clarified. When will the review actually begin, as presumably the end date for literature to be included will link to that.

Presumably the protocol will be registered, although maybe the authors are waiting for comments on this submission (and maybe this has already been done?). However, it would be good to do it before publication and include details.

Do the authors have any plans for dealing with a potentially large number of case reports of varying quality?

The rapidly evolving terminology in relation to vaping poses a problem, with terms like “disposables”, “puff bars”, Elf bars” , “pod mod” etc. The authors cite a 2023 publication but things have moved on. Any thoughts on how to deal with this? However, apart from this, the search strategy seems comprehensive.

VERSION 1 - AUTHOR RESPONSE

Author response to reviewer comments

This is a timely proposal, given the growing evidence of harm associated with e-cigarettes (including a recent review of their carcinogenic effects). And, as the authors note, given the strong evidence of harm associated with pre-operative smoking, the case is well made. One problem with following the increasingly strict formulaic approach to reviews is that the questions may be answered but leave as many questions as it answers. In this case, I think it would help to extract whatever information is in the studies on biological mechanisms. This information would need to be combined with evidence from studies that would not be included in this search, but would help to explain why any effects were seen. This could then be explored in the discussion section of the final review. But maybe this goes beyond the very formulaic guidelines for reviews.

- This is a useful suggestion, thank you. We have added a section to the data collection form (“Discussion points of interest”) to support this. If the studies report this data we will collect it and refer to it in our discussion.

I’m sure the editors will have checked that the protocol complies with all their requirements, such as describing planned or ongoing reviews, not including data, contain a Available rationale, and transparent methods. For the sake of completeness, I at least feel that the authors have done all this.

- Thank you - we have tried to be thorough and do feel we have met the requirements.

There are only a few things that may need to be clarified. When will the review actually begin, as presumably the end date for literature to be included will link to that.

- Thank you for this comment – the actual search date will be reported in the published review. Work will begin in early 2026.

Presumably the protocol will be registered, although maybe the authors are waiting for comments on this submission (and maybe this has already been done?). However, it would be good to do it before publication and include details.
- We have an entry for this scoping review on Open Science Framework (created Feb 2, 2026, 11:15 AM) https://osf.io/5fqc3/overview, and we will publish the protocol for this scoping review there. We will describe the date of submission to the journal, but we did not want to make a version of the protocol available online ourselves while it was being considered for publication by this journal. We have amended the manuscript to include the OSF registration in the methods.

Do the authors have any plans for dealing with a potentially large number of case reports of varying quality?
- We think it is important to give an overview of the literature as it stands. We will record and report on all case reports that meet our inclusion criteria, and provide a narrative overview of the case reports in our results. We will, however, only report in detail on the specifics of case reports which we feel bring useful information to the discussion (for example those which report respiratory complications).

The rapidly evolving terminology in relation to vaping poses a problem, with terms like “disposables”, “puff bars”, Elf bars” , “pod mod” etc. The authors cite a 2023 publication but things have moved on. Any thoughts on how to deal with this? However, apart from this, the search strategy seems comprehensive.
- We agree - the variable terminology is a potential limitation of all work investigating e-cigarette use. We mention this in the limitations section of our manuscript. The search was designed to include terms used within, and referenced by, the cited ontology to try to reduce this. MESH and EMTREE terms were used where applicable, as well as free text terms for each. Judicious use of "near" search operators also allowed us to use alternative terms such as "ENDS" or "ANDS" which would otherwise have produced an impossibly large number of search results. We feel the less formal terms you suggest are likely to be found in sources other than the academic literature, and in our experience

such articles almost always include a clarifying statement which uses a more traditional term such as e-cigarette or vape, and will therefore be picked up by our search.
